# Extracellular Matrix Orchestration of Tissue Remodeling in the Chronically Inflamed Mouse Colon

**DOI:** 10.1016/j.jcmgh.2024.01.003

**Published:** 2024-01-09

**Authors:** Elisa B. Moutin, Joanna Bons, Giada Giavara, Filipe Lourenco, Deng Pan, Jordan B. Burton, Samah Shah, Mathilde Colombé, Philippe Gascard, Thea Tlsty, Birgit Schilling, Douglas J. Winton

**Affiliations:** 1 Cancer Research UK Cambridge Institute, Li Ka Shing Centre, Cambridge, United Kingdom; 2 Buck Institute for Research on Aging, Novato, California; 3 Department of Pathology, University of California, San Francisco, California

**Keywords:** Inflammatory Bowel Disease, DIA Proteomics, Cell-Matrix Interactions, Proteoglycans

## Abstract

**Background & Aims:**

Chronic inflammatory illnesses are debilitating and recurrent conditions associated with significant comorbidities, including an increased risk of developing cancer. Extensive tissue remodeling is a hallmark of such illnesses, and is both a consequence and a mediator of disease progression. Despite previous characterization of epithelial and stromal remodeling during inflammatory bowel disease, a complete understanding of its impact on disease progression still is lacking.

**Methods:**

A comprehensive proteomic pipeline using data-independent acquisition was applied to decellularized colon samples from the *Muc2* knockout (*Muc2*^*KO*^) mouse model of colitis for an in-depth characterization of extracellular matrix remodeling. Unique proteomic profiles of the matrisomal landscape were extracted from prepathologic and overt colitis. Integration of proteomics and transcriptomics data sets extracted from the same murine model produced network maps describing the orchestrating role of matrisomal proteins in tissue remodeling during the progression of colitis.

**Results:**

The in-depth proteomic workflow used here allowed the addition of 34 proteins to the known colon matrisomal signature. Protein signatures of prepathologic and pathologic colitic states were extracted, differentiating the 2 states by expression of small leucine-rich proteoglycans. We outlined the role of this class and other matrisomal proteins in tissue remodeling during colitis, as well as the potential for coordinated regulation of cell types by matrisomal ligands.

**Conclusions:**

Our work highlights a central role for matrisomal proteins in tissue remodeling during colitis and defines orchestrating nodes that can be exploited in the selection of therapeutic targets.

## Graphical abstract

**Figure:**
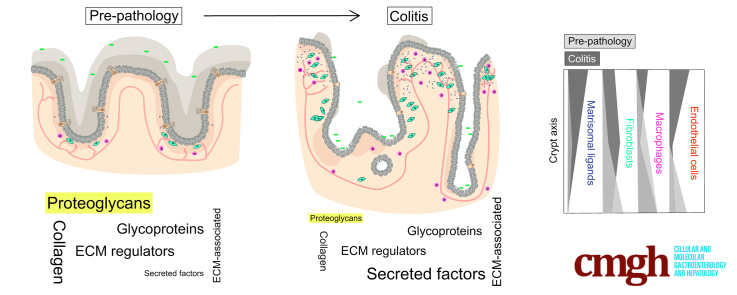

**Summary**

A novel proteomics methodology characterized extracellular matrix remodeling in colitis. Known changes in cell populations accompanying colitic disease were orchestrated by a subset of matrisomal proteins, defining interaction networks that can be exploited in disease prevention strategies.

Chronic inflammation generates compositional and adaptive changes at the cellular and molecular levels. Resulting tissue remodeling ultimately impairs epithelial function, leading to diverse disease states such as chronic liver disease, gastritis, or colitis, which are debilitating and life-altering conditions associated with an increased risk of developing cancer.^1^ Characterization of these chronic illnesses largely has been cell-centric, describing epithelial and stromal cell adaptations while viewing the extracellular matrix (ECM) and its associated proteins—the matrisome—as a supporting scaffold.

The matrisome comprises core ECM constituents (collagens, glycoproteins, proteoglycans, and polysaccharides), as well as associated regulators and secreted factors.^2^ It is produced by resident cell types that are found in unique combinations in different tissues.^3^ Evidence for an instructive capacity of the ECM originally came from studies in which separation of embryonic epithelium and mesenchyme before heterotypic recombination revealed that the mesenchyme was responsible for tissue differentiation.^4^ Subsequently, many studies have established that the matrisome is dynamically maintained and associated with varied cellular responses, including cell growth and differentiation through direct ligand-receptor interactions.^5^ Furthermore, it dictates biomechanical properties and is able to sequester and regulate the availability of cytokines and growth factors.^6^ More recently, embryonic precursor cells seeded in complete ECM scaffolds derived from decellularized tissues were found to differentiate into epithelia of the same tissue type as that from which the scaffold was derived.^7^, ^8^, ^9^

The relationship whereby the matrisome dynamically regulates the biology of the cells from which it arises has been termed *dynamic reciprocity*.^10^ During chronic inflammation, excessive degradation and inappropriate deposition of the ECM by proteases prevents restoration of normal matrix composition. This process, termed *fibrosis*, is linked to increased cancer risk..^11^^,^^12^ The ECM has been shown to influence all the hallmarks that typify development of cancer, and its remodeling is considered central to cancer initiation as a result of chronic inflammation.^13^^,^^14^, ^15^

Ulcerative colitis (UC) is a chronic inflammatory disorder prone to remission and relapse, with a complex and not yet entirely understood etiology involving both genetic and environmental factors.^16^ UC is defined by colonic mucosal inflammation associated with reduced quality and quantity of the mucins that make up the intestinal mucus layer, impaired epithelial barrier function, and engagement with both innate and adaptive immune systems.^17^^,^^18^ UC patients are at a greater risk of developing colitis-associated colorectal cancer, which increases with the duration and severity of disease, reaching 18% by 30 years.^19^^,^^20^

The complexity of tissue response and disease escalation in UC, as well as technological barriers in quantifying protein changes in fractions with low solubility/low abundance, have meant that the full repertoire of matrisomal changes as well as how and when these orchestrate cellular adaptation and recruitment in UC have not been appreciated. Recently, label-free mass spectrometry–based proteomic strategies based on comprehensive data-independent acquisition (DIA)^21^, ^22^, ^23^ have been used to identify proteins in ECM-enriched cancer tissue samples and provided deep, accurate, and reproducible quantification.^24^ Here, we apply this unbiased approach to a murine model of colitis to characterize alterations in matrisomal protein expression. Network analyses define how ECM changes converge on key signaling nodes in both prepathologic and pathologic states to identify vulnerabilities for therapeutic and preventive interventions.

## Results

### Experimental Approach to Investigate ECM Remodeling in Colitis

**Figure 1:**
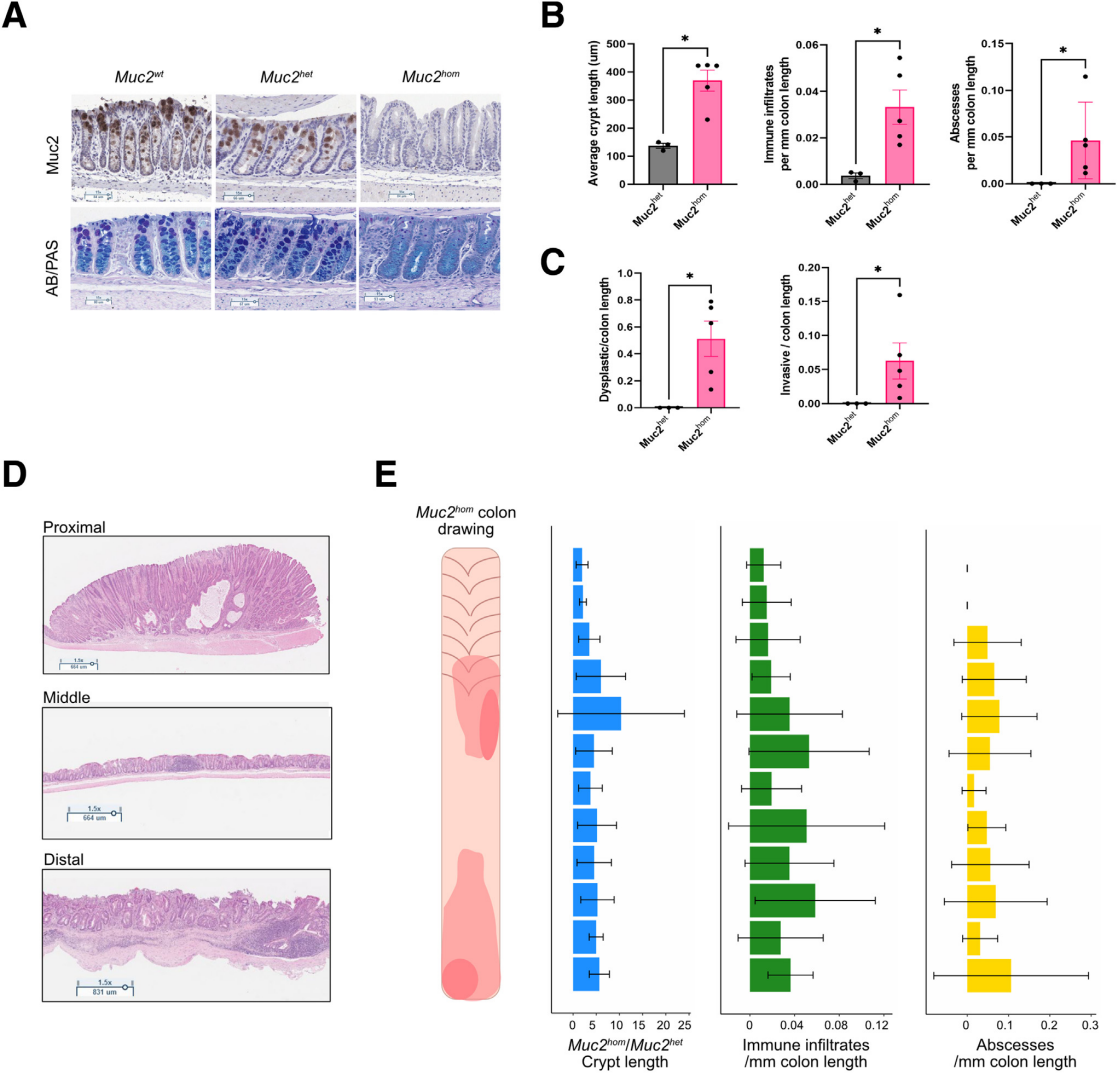
**Characterization of the *Muc2***^***KO***^**mouse model of colitis reveals spatial differences in pathology.** (*A*) Immunohistochemistry images for Muc2 (*top*) and Alcian blue (AB)/periodic acid–Schiff (PAS) (*bottom*), showing Muc2 deletion and hindered mucin production in *Muc2*^*hom*^ compared with *Muc2*^*wt*^ and *Muc2*^*het*^ mouse colon. (*B*) Bar plots showing average crypt length (*left*), as well as the number of immune infiltrates (*middle*) and abscesses (*right*) per millimeter in the *Muc2*^*hom*^ compared with *Muc2*^*het*^ colon (N = 5 *Muc2*^*hom*^ mice; median survival, 5.3 mo; N = 3 *Muc2*^*het*^ mice; median survival, 4.5 mo). Statistical significance was determined using the Mann–Whitney test, ∗*P* < .04. (*C*) Bar plots showing presence of preneoplastic hallmarks in the *Muc2*^*hom*^ colon. Dysplasia (*left*) and invasive length (*right*) are quantified as the proportion of respective pathology over the colon length, with E-cadherin+ glands crossing the muscularis mucosae considered as invasive. (*D*) Representative H&E images showing the spatial aspect of pathology along the mature *Muc2*^*hom*^ mouse colon. Proximal and distal regions are commonly inflamed, whereas the middle colon remains histologically normal. (*E*) Mature *Muc2*^*hom*^ colon drawing and histograms representing the spatial heterogeneity seen in crypt length, number of immune infiltrates, and abscesses along the length of the colon (N = 5 *Muc2*^*hom*^ mice used, normalization to N = 3 *Muc2*^*het*^ mice). Quantification was performed over 12 colonic regions as shown on the y-axis; error bars = 95% CI).

Germline genetic knockout of the *Muc2* gene in mice has been described previously as causing colitis that arises from impaired barrier function.^25^^,^^26^ This model was assessed phenotypically and mice with homozygous deletion of Mucin 2 (*Muc2*^*hom*^) were confirmed to show stochastic onset of colitis (Figure 1*A* and *B*). Staining with both Alcian blue and periodic acid–Schiff showed a reduction in expression of both acid and neutral mucins, and the remaining staining showed that goblet cells were retained (Figure 1*A*). This mouse model faithfully recapitulated key features of human UC including crypt enlargement and hyperplasia, immune infiltration of the mucosa, and the presence of abscesses (Figure 1*B*), as well as preneoplastic features such as dysplasia and early invasive epithelial foci (Figure 1*C*). In mature *Muc2*^*hom*^ mice (age, 5–8 mo), these phenotypes were present regionally in both the proximal and distal colon, whereas the middle colon remained histologically normal (Figure 1*D* and *E*).

**Figure 2:**
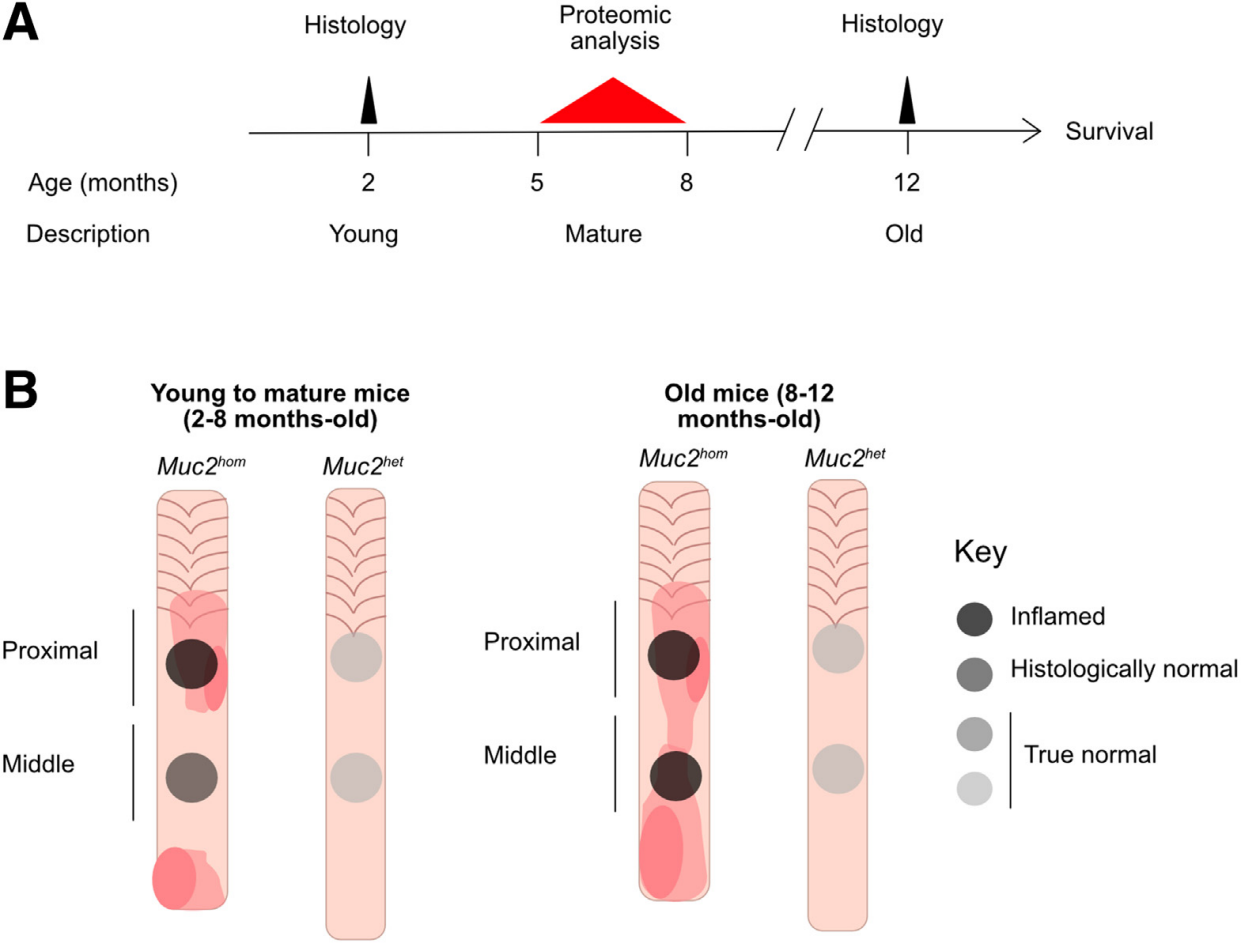
**Timeline and sample location for ECM proteomics.** (*A*) Timeline showing age of mice used for histopathologic analysis and ECM proteomics. (*B*) Scheme representing the location of samples used for ECM proteomics and associated level of pathology.

**Figure 3:**
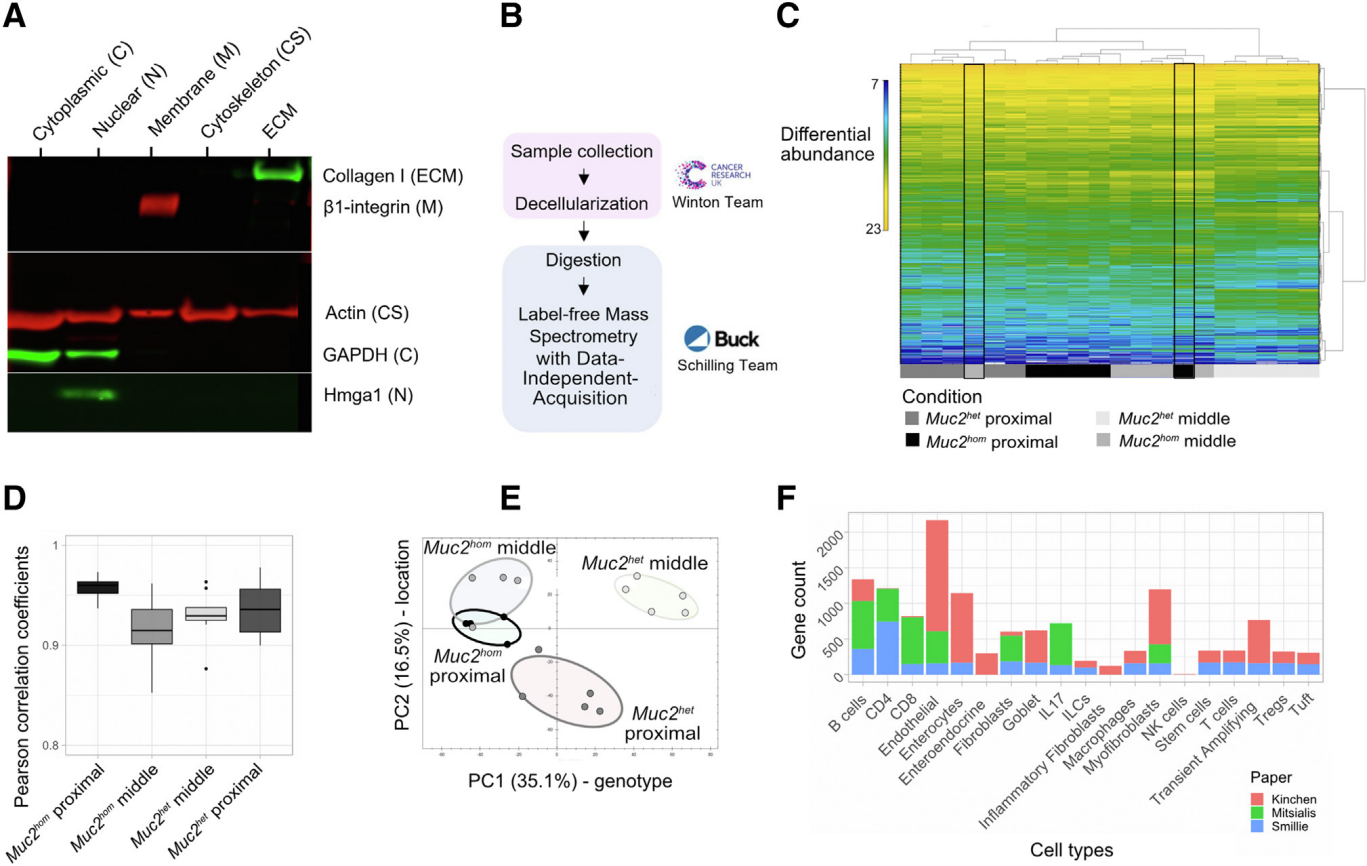
**Experimental approach to investigate ECM remodeling in colitis.** (*A*) Experimental outline for label-free MS on decellularized fractions of *Muc2*^*KO*^ mouse colon. (*B*) Western blot showing sequential purification of the extracellular tissue fraction. Glyceraldehyde-3-phosphate dehydrogenase (GAPDH) was used to probe for removal of cytoplasmic proteins, Hmga1 of nuclear proteins, β1-integrin of membrane proteins, and actin of cytoskeletal proteins. Enrichment for ECM is seen with appearance of a band for collagen 1. (*C*) Heatmap displaying identified and quantified protein groups for all samples, showing 2 out layers *outlined in black* that were removed from the analysis. (*D*) Box plots representing the distribution of Pearson correlation coefficients for samples in each condition. (*E*) Principal component analysis using the abundance of all quantified proteins between sample groups, where PC1 (x-axis) separates samples based on genotype and PC2 (y-axis) based on location along the colon. (*F*) Stacked bar plots showing the number of genes present in each extended cell type signatures, coming from each of 3 colon single-cell RNA sequencing published data sets (see details in Methods).

Samples for proteomic analysis were collected from 5 mature *Muc2*^*hom*^ mice aged until they presented with diarrhea, a symptom of active colitis, and culled when this was accompanied by other symptoms such as signs of pain (hunch, piloerection) and/or development of an anal prolapse. Tissue from 5 age-matched heterozygous (*Muc2*^*het*^*)* mice were used concurrently as controls (Figure 2*A*). The colon tissue was taken from proximal, actively inflamed regions and from middle uninvolved regions (Figure 2*B*). Samples were processed for enrichment of ECM proteins by sequential fractionation that was validated at each stage by Western blot (Figure 3*A*). The final protein fraction was solubilized, digested, and subjected to mass spectrometry (MS) analysis as described previously^24^ (Figure 3*B*). A spectral library was built directly from DIA MS/MS spectra and used to identify and quantify all proteins across all conditions. In total, 2328 protein groups were identified and quantified, with at least 2 unique peptides and a 1% false discovery rate. A clustering analysis based on all quantified protein groups led to removal of 2 outlier samples that clustered apart from their own sample group (Figure 3*C*). For the remaining samples, correlation coefficients for protein expression within the sample group was greater than 85% (Figure 3*D*). Principal component analysis revealed genotype and location along the colon as the main factors contributing to variance between sample groups (Figure 3*E*). Of the 2328 proteins identified, 953 (∼41% of the total protein fraction) were annotated as extracellular using the Gene Ontology term Cellular Component (Figure 1*A*). Within this subset, 125 proteins were identified as components or associated proteins of the colon matrisome (MatrisomeDB^2.0^),^27^ accounting for 70% of all annotated proteins in this data set. In addition, 37 identified proteins were part of the wider, pan-organ matrisome database, which now can be added to the known colon matrisomal signature (Supplementary Table 4).

### Proteomic Analysis Reflects Disease State

Proteins with expression altered significantly in colitis (defined as q-value < 0.05), identified from the comparison of proximal colonic regions of *Muc2*^*hom*^ inflamed and *Muc2*^*het*^ control mice, were assessed for alterations in biological processes using the molecular interaction database ConsensusPathDB.^28^ The analysis identified positive enrichment for ECM organization as well as different immune-related functions (Figure 1*B* and *C*), confirming ,the resulting data set being informative of ECM remodeling during colitis.

The analysis focused on proteins with low solubility, which, as reported previously, include cellular remnants such as keratins and desmosomal proteins that relate to overall tissue and cellular identity.^29^ To evaluate how protein alterations reflect cell population level changes accompanying inflammatory disease, differentially expressed proteins (defined as q-value < 0.05) from the comparison of *Muc2*^*hom*^ and *Muc2*^*het*^ proximal colons were assigned to specific cell types, using previously described signatures^30^, ^31^, ^32^ (see Methods and Figure 3*F*). This analysis identified enrichment for immune cell types and underrepresentation of epithelial secretory cells, notably goblet cells, reflecting well-known features of colitis (Figure 1*D* and *E*). These findings indicated that proteomic analysis of the extracellular protein fraction captured the complexity of tissue changes associated with disease state at the level of both biological processes and cell type identity.

### Deriving a Colitis Signature

To derive a robust colitis signature, we selected the top 200 proteins with the highest significance for differential expression in the proximal colon of *Muc2*^*hom*^ vs location-matched *Muc2*^*het*^ samples (Supplementary Table 3). Many up-regulated proteins were associated with specific immune cell types (*e.g.*, eosinophils peroxidase, myeloperoxydase or mast cell protease 1) or immune function, such as Immunoglobulin heavy constant alpha (Figure 1*F*). Included in this signature was also the down-regulation of small leucine-rich proteoglycans (SLRPs) asporin and osteoglycin, both present in the subset of proteins presented in Figure 1*F*, as well as decorin (Dcn), present in the extended inflammation signature Supplementary Table 5. SLRPs are known to bind and alter the properties of collagens.^33^ At the level of protein groups, proteins classified as proteoglycans showed the most dramatic change in expression, with the majority being down-regulated with inflammation (Figure 1*G*).

### Midcolon in Mature *Muc2*^*hom*^ Mice Displays a Prepathologic State

**Figure 5:**
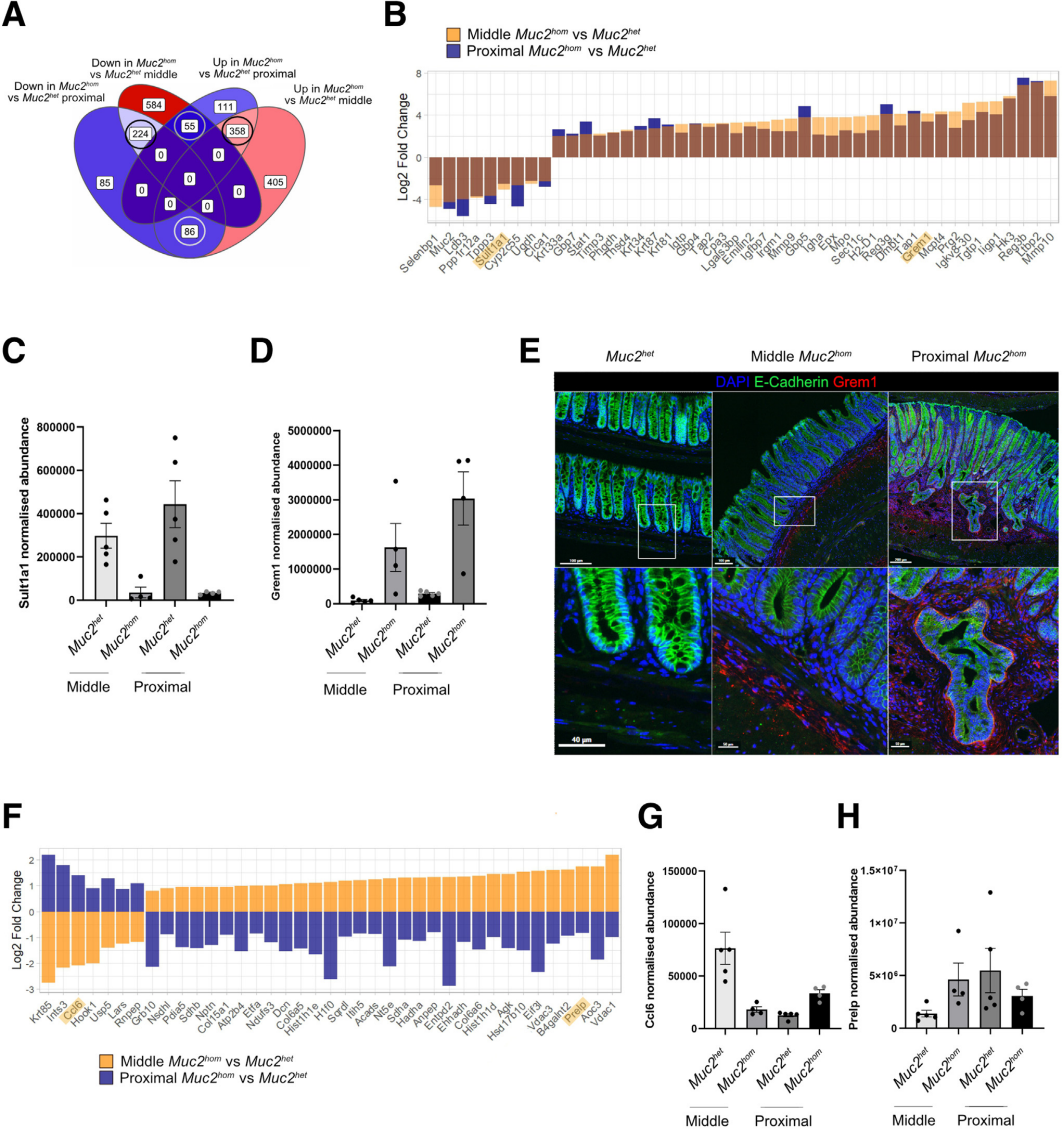
**Midcolon regions in mature *Muc2***^***KO***^**mice are in a prepathologic state.** (*A*) Venn diagram showing overlap in proteins up-downregulated and down-regulated (q-value < 0.05) in proximal and middle mature *Muc2*^*hom*^ mouse colon (inflamed and histologically normal, respectively, N = 4 samples per condition) compared with location-matched *Muc2*^*het*^ control samples (N = 5 for each location). Proteins sharing the same direction of change in both comparisons are *circled in grey* (see Supplementary Table 5), proteins showing the opposite direction of change are *circled in black* (see Supplementary Table 5). Fill color from blue to red: lower to higher number of proteins in each subset of the Venn diagram. (*B*) Bar plot showing a subset of proteins extracted from the shared signature (q-value < 0.01, log2 fold change >1 or <-1). (*C* and *D*) Highlighted proteins are shown. (*C*) Bar plots showing normalized protein abundance for sulfotransferase family 1A member 1 (Sult1a1), down-regulated in both proximal and middle mature *Muc2*^*hom*^ compared with location-matched *Muc2*^*het*^ control samples. (*D*) Bar plots representing normalized protein abundance for gremlin (Grem1), showing a similar pattern to Sult1a1, with a stronger sequential up-regulation from mid- to proximal *Muc2*^*hom*^ mouse colon. (*E*) Representative immunofluorescence images of Grem1 (in red) in *Muc2*^*het*^, midcolon *Muc2*^*hom*^, and proximal *Muc2*^*hom*^ tissue. 4′,6-diamidino-2-phenylindole (DAPI) marks nuclei, and E-cadherin marks colonic epithelial cells. *Bottom*: Magnification of the *white boxes* in the *top panel*. (*F*) Bar plot showing a subset of proteins showing opposing signatures in middle and proximal *Muc2*^*hom*^colon samples(q-value < 0.05, log2 fold change >1 or <-1). (*G* and *H*) Highlighted proteins are shown. (*G*) Bar plots showing normalized protein abundance for chemokine (C-C motif) ligand 6 (Ccl6), down-regulated in *Muc2*^*hom*^ midcolon and up-regulated in *Muc2*^*hom*^ proximal colon compared with *Muc2*^*het*^ control samples. (*H*) Normalized protein abundance for proline and arginine-rich end leucine-rich repeat protein (Prelp), up-regulated in Muc*2*^*hom*^ midcolon and down-regulated in *Muc2*^*hom*^ proximal colon compared with *Muc2*^*het*^ control samples.

Comparison of the midcolonic regions from *Muc2*^*hom*^ and *Muc2*^*het*^ mice identified 1712 differentially expressed proteins (Figure 5*A*). Of these, 721 were also differentially expressed in the proximal colonic region of *Muc2*^*hom*^ mice that showed signs of active colitis (Figure 5*A*). The similarity between proximal inflamed and middle histologically normal colon samples was apparent in the principal component analysis, where those samples clustered together (Figure 3*E*).

**Figure 6:**
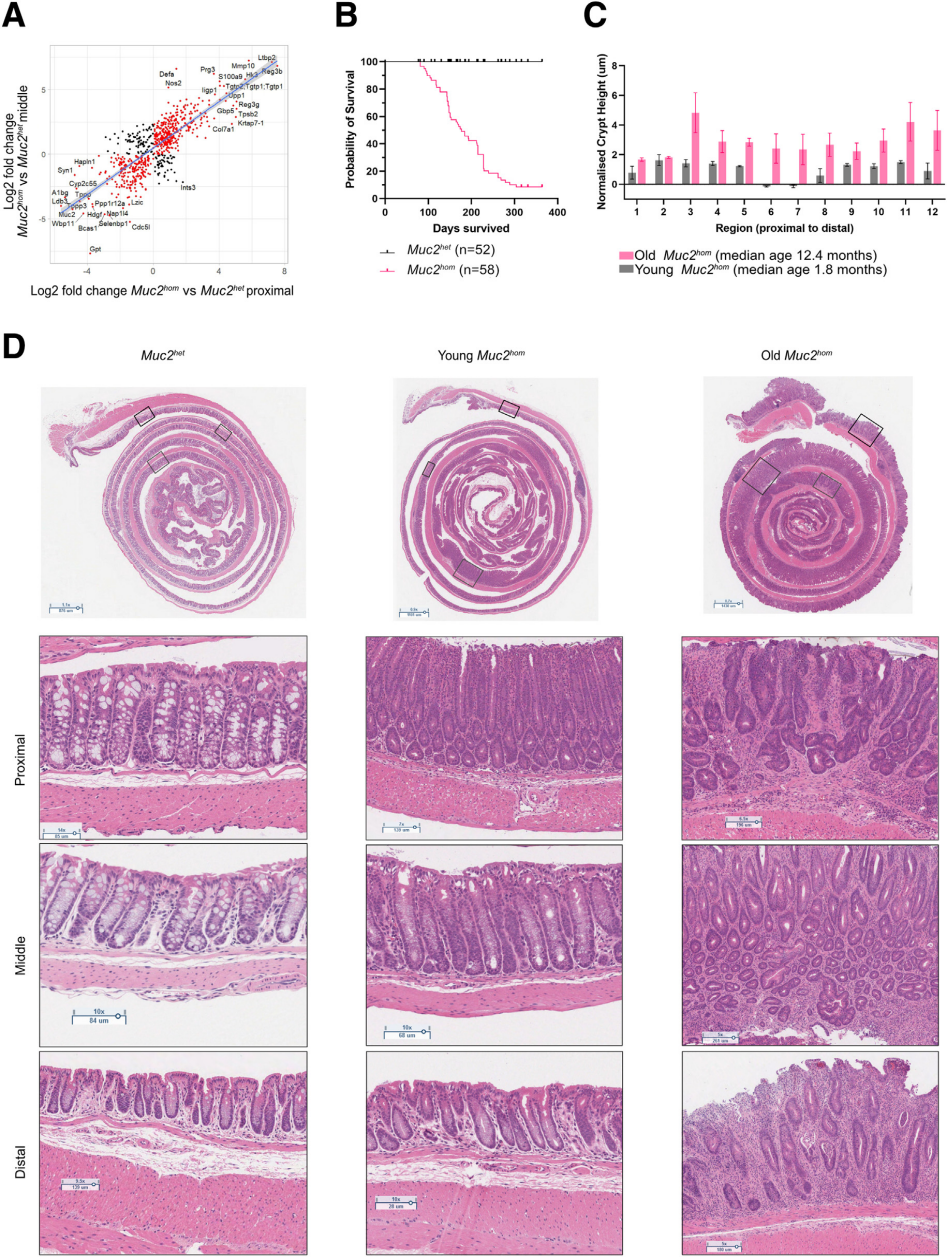
***Muc2***^***KO***^**mice evolve from discrete to pancolitis with age.** (*A*) Dotplot showing log2 fold changes for each protein expressed differentially in *Muc2*^*hom*^ midcolon (y-axis) and proximal (x-axis) samples compared with location-matched *Muc2*^*het*^ colon sample (q-value < 0.05). (*B*) Survival analysis showing heterogeneity in *Muc2*^*hom*^ mice survival, from 100 days up to 1 year old (study end point). (*C*) Bar plot showing crypt height for young (*graybars*; median age, 1.8 mo) and old (*pink bars*; median age, 12.4 mo) *Muc2*^*hom*^ mice, normalized to age-matched *Muc2*^*het*^ controls (NFig. 4 = 3 mice for each condition). (*D*) Representative H&E pictures of the whole swiss rolled colon (top) and specific colonic regions in Muc2het, (left) young *Muc2*^*hom*^(middle) and old *Muc2*^*hom*^ mice (right), showing development of pathology in the proximal colon in young *Muc2*^*hom*^ mice, which extends to the whole colon length in old *Muc2*^*hom*^ mice. Boxes indicate magnified regions.

Comparing differentially expressed proteins in proximal and middle colon samples from *Muc2*^*hom*^ (compared with location-matched *Muc2*^*het*^ samples) revealed a linear relationship with a positive correlation, showing that shared alterations also follow the same direction of change (Figure 6*A*). We hypothesized that the substantial overlap in the number of proteins sharing the same direction of change might be explained by 2 opposing interpretations. First, it may reflect stable adaptive changes accompanying loss of *Muc2* expression. Alternatively, it could indicate an early prepathologic stage in the development of inflammatory disease. To discriminate between these possibilities, a cohort of *Muc2*^*hom*^ mice were maintained with colitis symptoms (median survival, 12 mo; range, 11.7–12.6 mo) and analyzed for inflammation along the length of the colon (Figure 2). These older animals showed an overall increase in pathology-related metrics, as well as a trend toward pancolitis (Figure 6*B* and *C*). This observation supported that the middle region of the colon in mature *Muc2^hom^* mice constituted a prepathologic state.

A shared representative signature was derived from proteins expressed differentially in the comparison of inflamed and prepathologic *Muc2*^*hom*^ vs location-matched *Muc2*^*het*^ samples and that shared the same direction of change (Figure 2*A* and *B* and Supplementary Table 2). Sulfotransferase family 1A member 1 (Sult1a1), a protein involved in small-molecule metabolism and previously found to be down-regulated in DSS colitis,^30^ was down-regulated in both prepathologic and inflamed states (Figure 2*B* and *C*). In contrast, Gremlin 1 (Grem1) expression increased incrementally in the respective states (Figure 2*B* and *D*). Notably, immunohistochemistry analysis showed that although Grem1 expression was restricted to the basement membrane in the prepathologic state, it extended to the mucosa in inflamed tissues (Figure 2*E*).

Because the midcolonic mucosa remained histologically normal in 5- to 8-month-old *Muc2*^*hom*^ mice on initial presentation, we speculated that the observed molecular changes may illustrate an adaptation of the epithelium to inflammatory signals. Proteins were identified that were differentially expressed and changed in the opposite direction when comparing mid- and proximal inflamed regions of *Muc2*^*hom*^ mice to their respective control regions in *Muc2*^*het*^ samples (Figure 2*A* and *F* and Supplementary Table 2). Reciprocating patterns of expression were identified for ECM proteins including collagens (Col15a1, Col6a5, Col6a6) and SLRP proteoglycans such as proline and arginine-rich end leucine-rich repeat protein (Prelp) and Decorin (Dcn), as well as proteins implicated in mitochondrial function (Voltage-dependent anion-selective channel 1 and 3 or NADH-ubiquinone oxidoreductase 75 kDa subunit,) that all were up-regulated in midcolon and down-regulated in proximal inflamed colon (Figure 2*F* and *H*). Intriguingly, also showing this pattern was Vascular Adhesion Protein 1 (Vap-1), also known as Aoc3, a protein known to exist in tissue-bound and soluble forms and detected primarily in endothelial cells, adipocytes or serum, respectively. The tissue-bound form has been associated with tissue differentiation and ECM deposition whereas the soluble form has been shown to be proinflammatory and implicated in vascular diseases through involvement in leukocyte recruitment.^31^ Showing the opposing pattern (ie, down-regulated in midcolon and up-regulated in inflamed proximal) was chemokine ligand 6 (Figure 2*F* and *G*). Increased expression of this chemoattractant chemokine is consistent with the increased immune cell recruitment observed in inflammation.

### Differential Expression of Proteoglycans Distinguishes Pathologic States

Expression of 6 SLRPs in mid- and proximal inflamed regions of mature *Muc2*^*hom*^ mice was assessed relative to their respective control regions in *Muc2*^*het*^ samples. These canonical SLRPs^33^ play diverse roles in collagen deposition, bind to receptor tyrosine kinases, innate immune receptors, and modulate the transforming growth factor-β (TGF-β) signaling pathway.^34^

**Figure 7:**
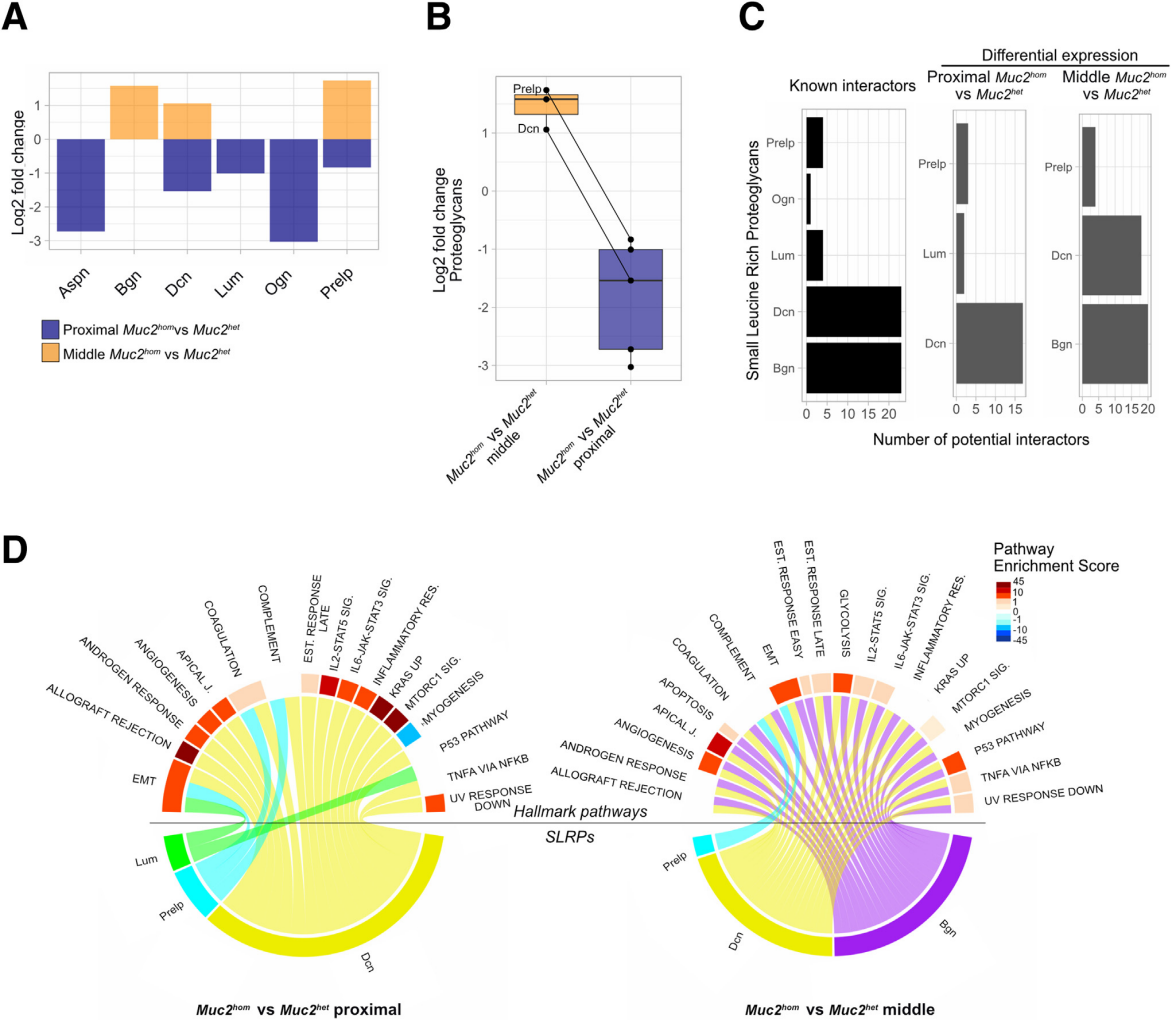
**Differential expression of proteoglycans distinguishes tissues in prepathologic and pathologic states.** (*A*) Bar plot showing distribution of log2 fold changes for Small Leucine Rich Proteoglycans (SLRPs) found significantly differentially expressed in the comparison of middle *Muc2*^*hom*^ vs *Muc2*^*het*^ and/or proximal *Muc2*^*hom*^ vs *Muc2*^*het*^ samples (q-value < 0.05). (*B*) Box plots representing differential protein expression as in panel *A*, showing down-regulation from the comparison of middle *Muc2*^*hom*^ vs *Muc2*^*het*^ to proximal *Muc2*^*hom*^ vs *Muc2*^*het*^ for SLRPs identified in both comparisons. Dcn and proline and Prelp are highlighted as candidates up-regulated in the middle *Muc2*^*hom*^ vs *Muc2*^*het*^ and down-regulated in proximal *Muc2*^*hom*^ vs *Muc2*^*het*^. (*C*) Bar plots showing the number of known interactors to SLRPs identified (*left*, in black) and those differentially expressed in the comparison of proximal *Muc2*^*hom*^ vs *Muc2*^*het*^ or middle *Muc2*^*hom*^ vs *Muc2*^*het*^ samples (*right*, in grey). (*D*) Circos plots displaying SLRPs on the bottom, differentially expressed in proximal *Muc2*^*hom*^ vs *Muc2*^*het*^ samples (*left*) or middle *Muc2*^*hom*^ vs *Muc2*^*het*^ samples (*right*). SLRPs are mapped to hallmark pathways (*top*) involving their known interactors, enriched (see Methods), either in proximal *Muc2*^*hom*^ vs *Muc2*^*het*^ samples (*left*) or middle *Muc2*^*hom*^ vs *Muc2*^*het*^ samples (*right*).

All identified SLRPs, in particular Prelp and Dcn, were down-regulated with inflammation whilst up-regulated in midcolon, illustrating a clear distinction in differential proteoglycan expression associated with early (prepathologic) and advanced pathologic states (Figure 7*A* and *B*). The Bader Laboratory CellCellInteraction database^35^ was used to explore the consequences of altered SLRP expression by identifying differentially expressed interactors in both states. Dcn and biglycan (Bgn) presented with the highest number of known interactors (Figure 7*C*). Pathway analysis based on differentially expressed SLRP interactors (see Methods) showed a strong enrichment for inflammation-related pathways such as Inflammatory Response and Allograft Rejection in the inflamed proximal but not the midcolon of *Muc2^hom^* mice (Figure 7*D*). Interestingly, an enrichment for the p53 pathway was observed in the midcolon of *Muc2^hom^* mice, contrasting with an up-regulation of the Kras pathway in inflamed proximal samples. Heightened p53 activity in midcolon was accompanied by positive enrichment for apoptosis, suggesting a possible role in control of cellular integrity. No interactors of SLRPs could be mapped to the p53 or apoptosis pathway in proximal inflamed samples, suggesting that this pathway might be down-regulated to allow for cell proliferation necessary for tissue repair. Taken together, these findings highlight the central role that down-regulation of proteoglycans plays in the progression of chronic inflammation.

### Matrisomal Orchestration of Tissue Remodeling in Colitis

**Figure 4:**
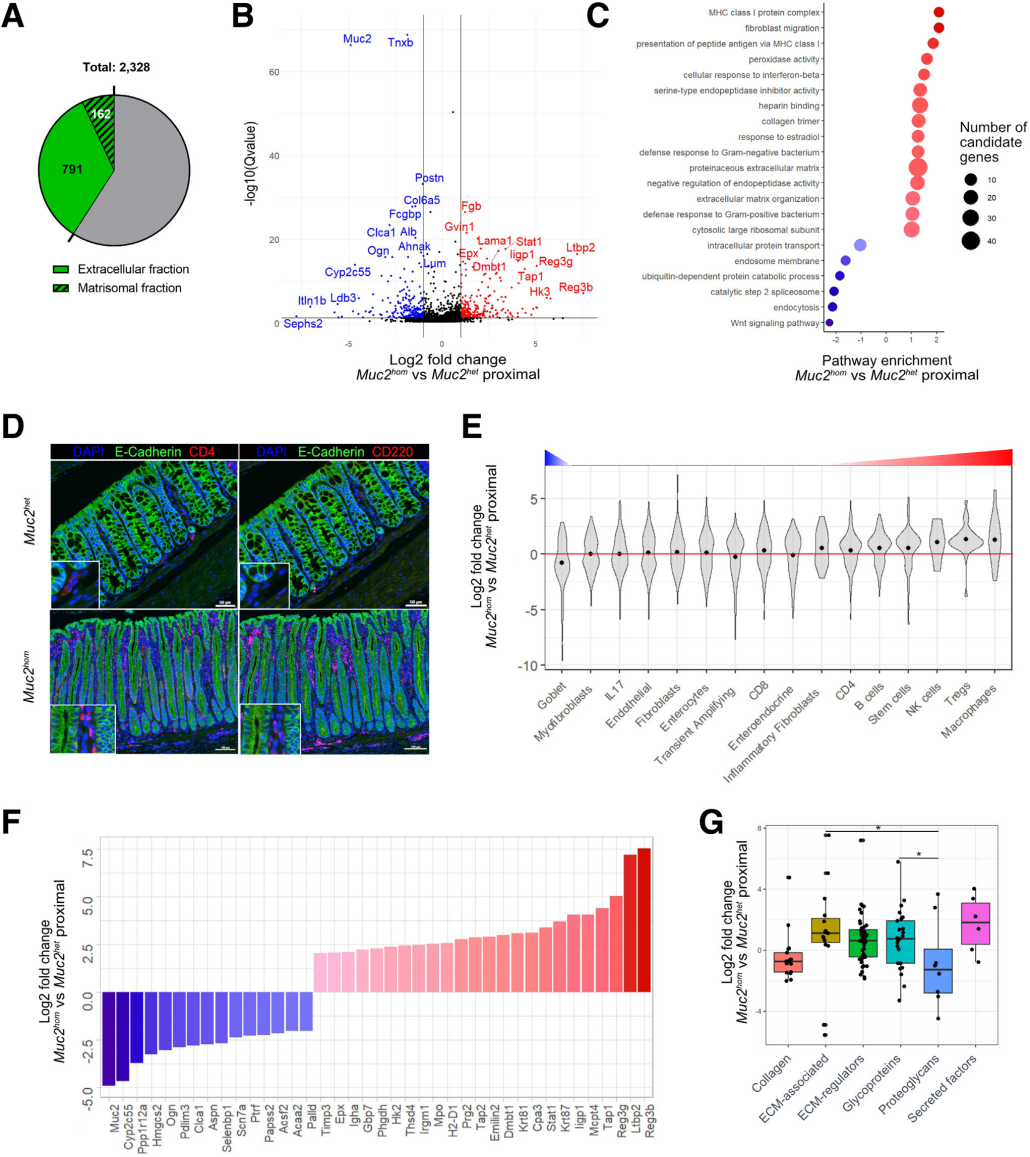
**Proteomic analysis of an extracellular matrix–enriched tissue fraction reflects disease state.** (*A*) Venn diagram showing the proportion of proteins identified through DIA-MS classed as extracellular based on Gene Ontology terms (953; 40.9% of the total protein fraction) and the proportion of matrisomal proteins identified (162; amounting to 5% of the total protein fraction). (*B*) Volcano plot showing differentially expressed proteins in the proximal colon of *Muc2*^*hom*^ (N = 4) vs *Muc2*^*het*^ control (N = 5) mice. Red indicates significant up-regulation and blue indicates significant down-regulation (log2 fold change >1 or <-1; q-value < 0.05). (*C*) Dot plot showing ConsensusPathDB output,assessing pathway enrichment for *Muc2*^*hom*^ vs *Muc2*^*het*^ proximal colon samples. The top significantly enriched pathways are represented (*P* < .01). (*D*) Immunofluorescence pictures showing enrichment for CD4+ T cells (*left*) and B220/CD45R+ B cells (*right*) in *Muc2*^*hom*^ compared with Muc*2*^*het*^ proximal colon (4′,6-diamidino-2-phenylindole [DAPI] marking nuclei, and E-cadherin marking colonic epithelial cells). (*E*) Violin plots showing the distribution of protein log2 fold changes in *Muc2*^*hom*^ compared with Muc*2*^*het*^proximal samples (q-value < 0.05), where proteins are mapped to different cell type signatures (details in Methods). Positive enrichment is indicated by the median (*black dot*) above the red line, and negative enrichment when under this line. (*F*) Protein differential expression signature was extracted from the comparison of *Muc2*^*hom*^ compared with Muc*2*^*het proximal*^ samples (see Supplementary Table 2). A subset is shown here where log2 FC >2 or <-2, and q-value < 5 × 10e-7. (*G*) Box plots showing differential expression of proteins in *Muc2*^*hom*^ compared with Muc*2*^*het*^ proximal samples (q-value < 0.05), where ECM proteins are classed into categories. ∗*P* < .05, pairwise *t* test.

**Figure 8:**
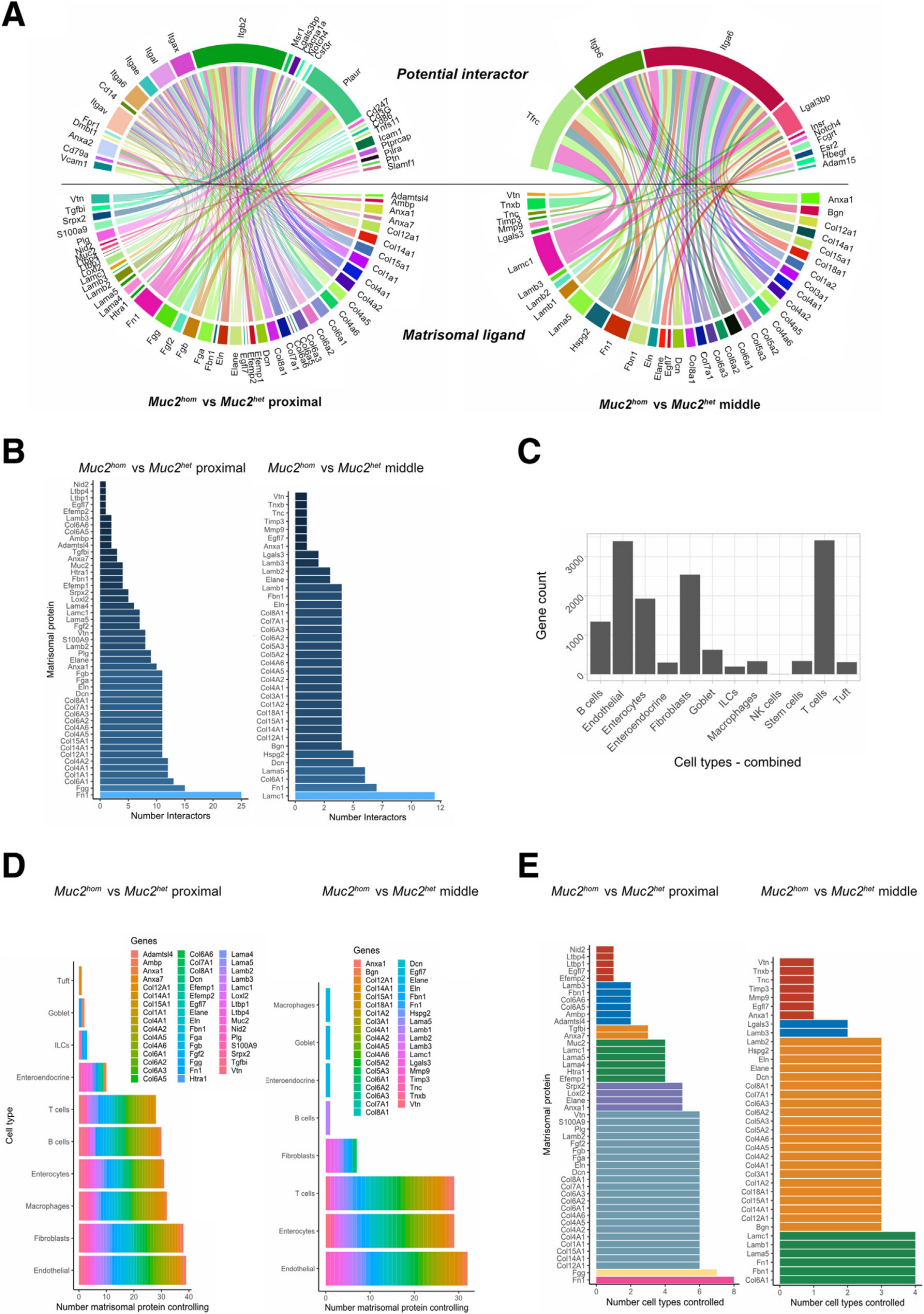
**Building networks representing matrisomal orchestration of cell type remodeling.** (*A*) Circos plot showing differentially expressed matrisomal ligands (*bottom*, q-value < 0.05) and their known interactors expressed differentially at the transcriptomic level (*top*, adjusted *P* value < .05), in proximal Muc*2*^*hom*^ (*left*) and middle *Muc2*^*hom*^ (*right*) compared with location-matched *Muc2*^*het*^ control samples. (*B*) Histograms showing the number of receptors expressed differentially at the transcriptomic level (x axis, adjusted *P* value < .05), for each matrisomal ligand differentially expressed in the comparison of proximal Muc*2*^*hom*^ vs *Muc2*^*het*^ (*left*) or middle *Muc2*^*hom*^ vs *Muc2*^*het*^ (*right*). (*C*) Bar plot representing the number of genes present in each combined cell type signatures, combining discrete cell types (see Methods). (*D*) Histogram showing the number (x-axis) and name of matrisomal proteins (as described in panel *B*) controlling each cell type present on the y-axis (combined cell type signature). (*E*) Histogram showing the number of cell types (combined cell type signature) controlled by each matrisomal protein as described in panel *B*.

**Figure 9:**
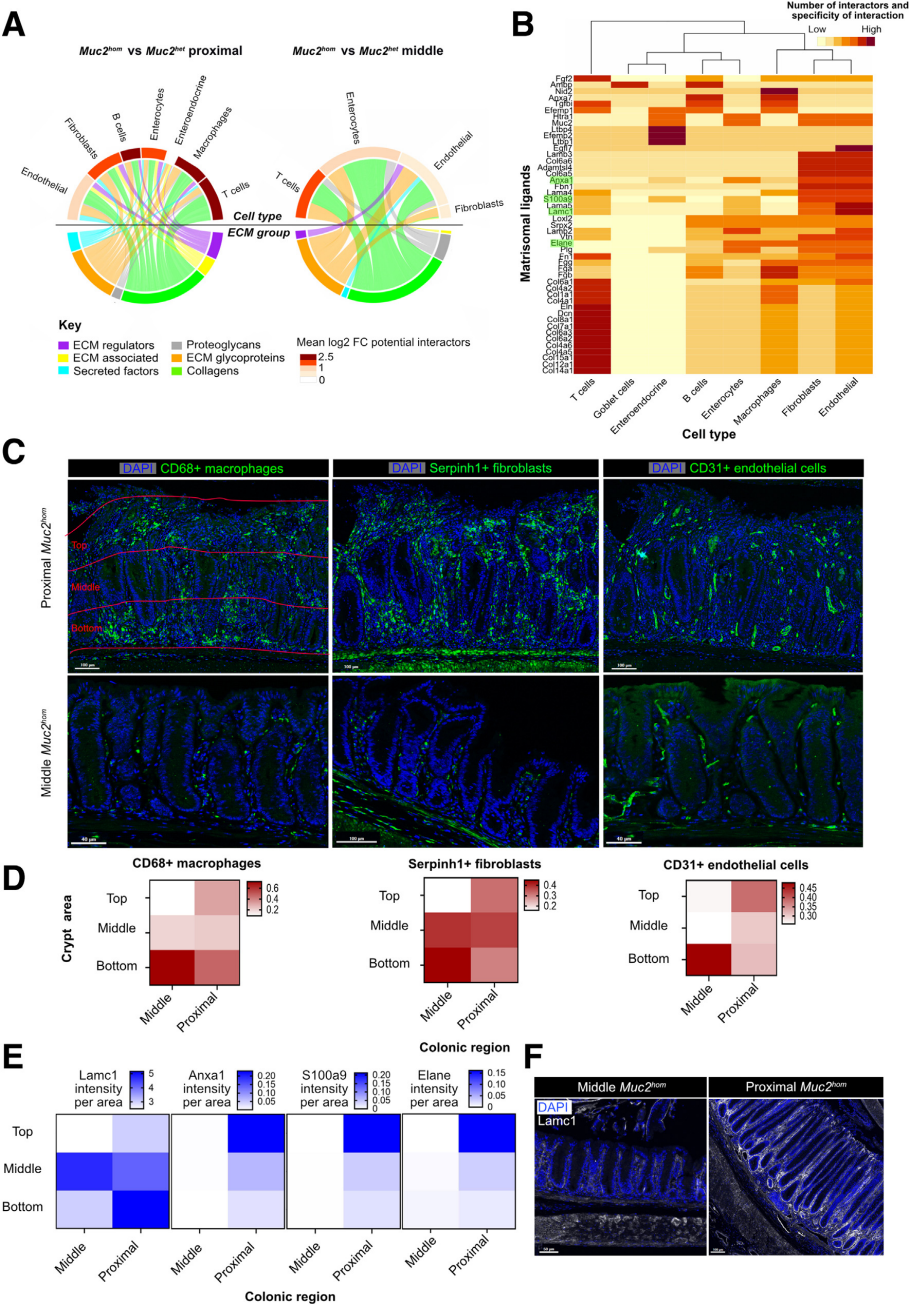
**Matrisomal orchestration of tissue remodeling in colitis.** (*A*) Circos plot showing matrisomal ligands differentially expressed (q-value < 0.05) in proximal *Muc2*^*hom*^ vs *Muc2*^*het*^ (*left*) or middle *Muc2*^*hom*^ vs *Muc2*^*het*^ (*right*), organized by groups (*bottom*) linked to cell types (*top*) containing receptors with which they are known to interact and are expressed differentially at the transcriptomic level (adjusted *P* value < .05, see Methods for details). The heat on the *top panel* represends mean log2 fold change for interactors to matrisomal ligands mapping to each cell type. (*B*) Heatmap showing matrisomal proteins part of the inflamed matrisome (y axis) and the cell types within which they have potential interactors (x-axis). The heat represents the number of interactors of each matrisomal ligand within each cell type, and is normalized per row, giving more power to unique interactions. (*C*) Immunofluorescence images showing the location of CD31+ endothelial cells (*left panel*, in red), CD68+ macrophages (*middle panel*, in yellow), and Serpinh1+ fibroblasts (*right panel*, in green) in proximal and middle *Muc2*^*hom*^ colon. (*D*) Heatmaps showing the percentage of positive cells for each of the 3 cell types in 3 regions of the same area along the crypt axis (*bottom*, *middle*, and *top*) in proximal and middle *Muc2*^*hom*^ colon (N = 3). (*E*) Heatmaps showing the expression of 4 ECM ligands in proximal and middle *Muc2*^*hom*^ colon, expressed as intensity per area (N = 3). (*D*) Representative immunofluorescence image showing the distribution of Lamc1 along the crypt axis in proximal and middle *Muc2*^*hom*^ colon. DAPI, 4′,6-diamidino-2-phenylindole.

Next, to infer the orchestrating role of matrisomal proteins in tissue remodeling, the 162 matrisomal proteins identified (Supplementary Table 1, Figure 4*A*) were interrogated for differential expression in mature *Muc2*^*hom*^ mice, selecting only proteins annotated as ligands in the CellCellInteraction database.^35^ The same database was then was used to infer potential interactions using receptors found to be expressed differentially in *Muc2*^*hom*^ mice at the transcriptomic level. In inflamed proximal *Muc2*^*hom*^ tissue, 47 matrisomal proteins were identified as ligands to 27 differentially expressed receptors. In contrast, in midcolon, only 10 receptors and 37 matrisomal proteins were differentially expressed (Figure 8*A*, Supplementary Table 1), showing that the matrisomal regulome in prepathology maps to significantly fewer interactors than in inflamed tissue. For example, fibronectin (Fn1) interacted with 25 receptors in inflamed tissue, but with only 7 in prepathology (Figure 9*B*). Furthermore, the repertoire of differentially expressed integrins was reduced in prepathology. The only integrins engaging with matrisomal ligands were Integrin alpha and beta 6, compared with the inflamed state in which Integrin av, ae, al, az and b2 2 additionally were engaged. Taken together, this shows a greater complexity of regulation in overt inflammation compared to prepathology.

Receptors interacting with matrisomal ligands were then mapped to cell types to reveal the extent of the matrisomal contribution to regulation of tissue function in the context of colitis (Figure 9*C*). This analysis indicated that 10 cell types were potentially regulated by differentially expressed matrisomal proteins in inflamed tissue, compared with 8 in prepathology (Figure 9*D*). In both states, endothelial cells had the most connections to matrisomal proteins. Matrisomal regulation of macrophages and B cells appeared limited to inflamed tissue (Figure 5*A*). Proteins such as fibronectin (Fn1) were implicated in engagement of 8 different cell types in inflammation. In prepathology, engagement of 4 cell types was the maximum for any matrisomal protein (Figure 9*E*).

Finally, an integrated network was created to link differentially expressed matrisomal proteins to the cell types they regulate (Figure 9*A*). This network revealed greater involvement of ECM regulators and secreted factors in inflammation compared with prepathology, and more widespread tissue landscape alterations extending to include immune cell types such as B cells, T cells, and macrophages.

Hierarchical clustering was used to recognize similarities in the regulation of different cell types by matrisomal proteins altered in inflammation (Figure 9*B*). This analysis revealed a possible coordinated regulation of fibroblasts and endothelial cells by a subset of matrisomal proteins, including lysyl oxidase homolog 2 (Loxl2) and neutrophil elastase (Elane). Of note, the presence of matrisomal proteins such as S100 calcium-binding protein A9 (S100A9), plasminogen (Plg), and Loxl2 in this signature suggests a shared function in increasing collagen cross-linking (Figure 9*B*).

To gain direct evidence for this coordinated regulation by matrisomal ligands, the distribution of macrophages, fibroblasts, and endothelial cells along the colonic crypt axis was first compared using immunofluorescence in *Muc2*^*hom*^ prepathologic midcolon and inflamed proximal tissue (Figure 9*C* and *D*). In the former, CD31+ endothelial cells, CD68+ macrophages, and Serpin H family member 1 (Serpinh1)+ fibroblasts are located primarily in a region adjacent to the lower crypt epithelium and submucosa. With inflammation, fibroblasts and endothelial cells show a marked displacement toward the luminal surface at the top of the crypts, whereas macrophages largely remain in a more basal location. Next, the expression profile of 4 matrisomal ligands (Laminin subunit gamma 1 (Lamc1), Annexin 1, S100A9, and Elane) that are associated predominantly with fibroblast and endothelial regulation along the crypt axis was assessed similarly (Figure 4*B* and *E*). This revealed a trend for increased expression of these ligands in the same luminal region with inflammation. Of note, Lamc1 expression also was displaced toward the basal side of crypts (Figure 9*F*). These findings suggest that spatial changes in the frequency of fibroblasts and endothelial cells can be tracked to the ligands associated with their regulation. Together, these results define the orchestrating role of the matrisome in the development of colonic inflammatory disease.

## Discussion

Despite widespread acceptance of the fundamental role of the matrisome in regulating tissue homeostasis, as well as remodeling in chronic disease states such as colitis, relatively few studies have focused on understanding and integrating the full repertoire of matrisomal orchestration.^36^ Here, the development of label-free mass spectrometry (DIA-MS) approaches to quantify proteins in ECM-enriched samples provided the opportunity to build an integrated picture of matrisomal regulation in a mouse model that captures much of the pathology and dysfunction seen in UC patients.^25^^,^^26^^,^^37^ Importantly, the choice of model also enabled identification of ECM remodeling events and candidate pathways involved in early tissue adaptation to chronic inflammation before any histologic manifestation.

Combining protein fractionation with comprehensive and sensitive data-independent acquisition–mass spectrometry revealed the extensive tissue remodeling accompanying colitis and related it to mediators of the inflammatory phenotype. For example, within the colitis signature, latent TGF-β proteins are enriched and are responsible for directing latent TGF-β to extracellular matrix microfibrils, where it becomes bioavailable upon tissue remodeling to mediate both inflammatory and fibrotic responses seen both in the *Muc2*^*KO*^ model and in UC patients.^38^^,^^39^ In addition, the depth of this unbiased proteomic analysis allowed deconvolution of cell populations known to be associated with colonic inflammation in both murine models of colitis as well as in human disease, using approaches previously applied at the transcriptomic level using single-cell technologies.^40^, ^41^, ^42^

Many differentially expressed proteins were detected in the apparently histologically normal middle colon, which led to the finding that this tissue already had acquired a prepathologic state. This finding is reminiscent of recent observations in a delayed-onset model of induced colitis that identified protein changes before the onset of inflammatory disease.^36^ Analysis of colitis in older animals confirmed the development of a pancolitis, allowing interrogation of the proteomic data for adaptive responses that may restrain histologic manifestations of inflammation at earlier disease stages.

One class of proteins that may be involved in restricting inflammatory disease are SLRPs, which are increased in prepathology before their reduction in overt inflammation. Down-regulation of small leucine-rich proteoglycans is associated with poor outcomes in invasive breast cancer,^43^ and in chronic inflammation–associated cancers, including lung squamous cell carcinoma.^24^ Interrogating interactors of SLRPs differentially expressed in prepathology, we found evidence for increased p53 activity that was not seen in the inflamed state. This is in accord with previous studies showing that the proliferative phase of tissue regeneration requires the coordinated up-regulation of trophic pathways (as seen with Kras here) while down-regulating that of p53.^44^ Hence, the transient increased expression of SLRPs in the prepathologic state may coincide with the activation of a p53 checkpoint control that subsequently is lost as inflammatory disease develops, resulting in a corresponding increase in cancer risk.

Notably, the prepathologic state was associated with increased expression of many mitochondrial proteins. In inflammatory bowel disease patients, genetic risk factors affecting mitochondrial function have been identified and their altered metabolism proposed as causative in inflammatory disease.^45^, ^46^, ^47^ Recently, altered mitochondrial function has been shown to predict disease recurrence in Crohn’s disease, supporting a role for mitochondrial dysfunction developing before the onset of active inflammation.^48^

To uncover the contribution of the matrisome in orchestrating alterations in tissue ecology during colitis, an integrative approach was used to map ECM ligands to receptors with altered expression at the transcriptomic level. This ligand-receptor interaction analysis showed positive interactions between matrisomal proteins and T-cell, B-cell, and macrophage populations consistent with their expansion in inflamed tissues. Moreover, common matrisomal protein subsets could be associated with specific cell types such as fibroblasts and endothelial cells. For example, in the inflamed state, of the 38 proteins interacting with fibroblasts and 39 interacting with endothelial cells, only EGF-like domain protein 7 (Egfl7) is specific to endothelial cells. The other 38 proteins interact with both endothelial cells and fibroblasts. Spatial analysis showed a displacement of specific matrisomal ligands toward the top of crypts in inflamed regions, which was associated with a displacement of both cell types, illustrating a coordinated regulation driven by ECM remodeling accompanying colitis. Matrisomal ligands associated with remodeling of these cell types were produced by immune cells or inflammatory fibroblasts, representing an example of dynamic reciprocity in the context of colitis.

In contrast, in the prepathologic state, fewer matrisomal ligands and interactors are engaged and they are predicted to have an impact on a smaller number of cell types, primarily epithelial and endothelial cells. Therefore, assessing the prepathologic tissue landscape to determine how altered ECM composition engages with and regulates different cell types might provide new insights into how early tissue adaptations delay the subsequent onset of colitis. These observations will benefit from performing complementary studies of uninvolved mucosa in UC patients to establish if episodic recurrence of colitis in defined regions relates to local loss of protective mechanisms rather than an acquisition of susceptibility.

## Methods

### Mice

Animal care and procedures were performed at the Cancer Research UK Cambridge Institute Biological Resource Unit according to UK Home Office guidelines. Mice were of a C57BL/6 background. The *Muc2*^*KO*^ line used was described by Velcich et al.^25^ Genotyping of *Muc2*^*KO*^ mice was outsourced to Transnetyx (Cordova, TN).

#### Treatment of animals

The mice were housed under controlled conditions (temperature, 21°C ± 2°C; humidity, 55% ± 10%; 12-hour light/dark cycle) in a specific-pathogen free facility (tested according to the recommendations for health monitoring by the Federation of European Laboratory Animal Science Associations). Animals had unrestricted access to food and water.

### Histology

#### Immunohistochemistry/immunofluorescence

Mouse colons were opened longitudinally, fixed overnight in 4% paraformaldehyde, and processed for histology by conventional means. Sections were dewaxed and rehydrated, followed by heat-induced epitope-retrieval using 10 mmol/L Tri-sodium citrate buffer pH 6.0. Immunochemistry or immunofluorescence was performed as described previously.^49^ Antibodies used in immunohistochemistry were Muc2 (cat. sc-15334; Santa Cruz) and Ki67 (ab15580; Abcam). Antibodies used in immunofluorescence were E-cadherin (cat. 610182; BD Bioscience), B220/CD45R (cat. MAB1217; R&D Systems), Grem1 (cat. AF956; R&D Systems), CD68a (cat. 98941; Cell Signaling), Serpinh1 (cat. ab109117; Abcam), CD31 (cat. ab28364; Abcam), and Lamc1 (cat. ab233389; Abcam).

#### Spatial phenotyping

Fixed colons were Swiss-rolled luminal side in, starting from the proximal end. On-edge sections were cut at 3 different levels and stained with H&E. Immune hallmarks were scored and normalized along the colon length.

#### Image analysis

The Indica Laboratories HALO image analysis platform was used. The Indica Laboratories Area Quantification FL v.1.0 program was used for quantification of fluorescent staining (percentage of tissue stained and intensity of staining). The Indica Laboratories Highplex FL v.2.2.3 program was used for cell deconvolution based on 4′,6-diamidino-2-phenylindole nuclear expression and quantification of the percentage of cells expressing specific fluorescent markers. For spatial analysis along the crypt axis, layers of the same area were drawn on the bottom, middle, and top of the crypt, within which the analysis was performed.

### Extracellular Matrix Enrichment

#### Decellularization

Colon tissue was dissected from 6-month-old *Muc2*^*hom*^ mice and *Muc2*^*het*^ mice (n = 5 mice per genotype). After flushing with phosphate-buffered saline, 40–60 mg colon tissue pellets were flash-frozen and stored at -80°C. On the day of decellularization, pellets were homogenized using a Qiagen Lyser, before processing using a Compartmental Extraction Kit (2145; Millipore) as per the manufacturer’s protocol. This allowed extraction of extracellular matrix proteins through stepwise washes with salt solutions and detergents.

#### Western blot

Purification of each cellular compartment was tested by Western blot with antibodies for glyceraldehyde-3-phosphate dehydrogenase (cat. G8795; Sigma), High Mobility Group AT-Hook 1 (Hmga1) (cat. ab129153; Abcam), β1 integrin (cat. 610467; BD Bioscience), β-actin (cat. ab6276; Abcam), and collagen 1 (cat. PA5-95137; Invitrogen). One fifth of the extracellular protein pellet was dissolved in 30 uL of 4× sodium dodecyl sulfate (SDS) buffer supplemented with 100 mmol/L dithiothreitol (DTT). For other compartments, 10 uL sample was mixed with 10× lithium dodecyl sulfate (LDS) and 4× SDS.

### Label-Free Mass Spectrometry / Data Independent Acquisition

#### Chemicals

Liquid chromatography–MS grade acetonitrile (ACN), methanol, and water were obtained from Burdick & Jackson (Muskegon, MI). Reagents for protein chemistry, including SDS, ammonium bicarbonate, triethylammonium bicarbonate (TEAB), iodoacetamide, DTT, sequencing-grade endoproteinase Lys-C, and formic acid (FA) were purchased from Sigma-Aldrich (St. Louis, MO). Sequencing-grade trypsin was purchased from Promega (Madison, WI). Glycerol-free Peptide:N-glycosidase F was purchased from New England BioLabs (Ipswich, MA).

#### Solubilization of ECM proteins

The extracted ECM pellets were solubilized by agitation for 10 minutes in a solution containing 1% SDS and 50 mmol/L DTT, followed by sonication for 10 minutes, and, finally, heating at 85°C for 1 hour with agitation.

#### Protein digestion and desalting

Samples were solubilized with 4% SDS and 50 mmol/L TEAB at pH 8. Proteins were reduced with 20 mmol/L DTT (10 min at 50°C followed by 10 min at room temperature) and then alkylated with 40 mmol/L iodoacetamide (30 min at room temperature in the dark). Samples were acidified with a final concentration of 1.2% phosphoric acid, and diluted with 7 volumes S-trap buffer (90% methanol in 100 mmol/L TEAB, pH 8). Samples then were loaded onto the S-trap microspin columns (Protifi, Farmingdale, NY) and spun at 4000 × *g* for 10 seconds. The S-trap columns were washed with S-trap buffer twice at 4000 × *g* for 10 seconds each, before incubating the proteins with 250 ng sequencing-grade endoproteinase Lys-C in 50 mmol/L TEAB (pH 8) at 37°C for 2 hours. Then, 2.4 μg sequencing grade trypsin in 50 mmol/L TEAB (pH 8) for 1 hour at 47°C were added to the sample. After a 1-hour digestion at 47°C, the same amount of trypsin was added again and proteins were digested overnight at 37°C. Peptides were eluted sequentially with 50 mmol/L TEAB (pH 8), 0.5% FA in water, 50% acetronitrile, and 0.5% FA in water. After vacuum drying, samples were resuspended in 300 μL of 25 mmol/L ammonium bicarbonate in water, and spot-checked to ensure a pH of 7–8. Subsequently, 9 μL (4500 U) glycerol-free PNGase F were added, and samples were incubated for 3 hours at 37⁰C with agitation. This reaction was quenched with 10% FA in water for a final concentration of 1%, and spot-checked again to ensure a pH of 2–3. The quenched peptide samples were vacuum dried and resuspended in 20 μL of 0.2% FA, before desalting them using ZipTip (Merck, C5737) with 0.6 μL C_18_ resin (Sigma-Aldrich). Finally, samples were concentrated in a vacuum concentrator and resuspended in aqueous 0.2% FA containing indexed retention time (iRT) peptide standards (Biognosys, Schlieren, Switzerland).^50^

#### Mass spectrometric analysis

Liquid chromatography–tandem MS analyses were performed on a Dionex UltiMate 3000 system coupled to an Orbitrap Eclipse Tribrid mass spectrometer (both from Thermo Fisher Scientific, San Jose, CA). The solvent system consisted of 2% ACN, 0.1% FA in water (solvent A) and 98% ACN, 0.1% FA in water (solvent B). Proteolytic peptides were loaded onto an Acclaim PepMap 100 C_18_ trap column (0.1 × 20 mm, 5 μm particle size; Thermo Fisher Scientific) over 5 minutes at 5 μL/min with 100% solvent A. Peptides were eluted on an Acclaim PepMap 100 C_18_ analytical column (75 μm × 50 cm, 3 μm particle size; Thermo Fisher Scientific) at 0.3 μL/min using the following gradient of solvent B: 2% for 5 minutes, linear from 2% to 20% in 125 minutes, linear from 20% to 32% in 40 minutes, up to 80% in 1 minute, 80% for 9 minutes, and down to 2% in 1 minute. The column was equilibrated at 2% for 29 minutes (total gradient length = 210 min).

Every sample was acquired in data-independent acquisition (DIA) mode [20-22] using the following settings: full MS spectra were collected at 120,000 resolution (Automatic Gain Control (AGC) target: 3e6 ions, maximum injection time: 60 ms, 350–1650 m/z), and MS/MS spectra at 30,000 resolution (AGC target: 3e6 ions, maximum injection time: auto, Normalised Collision Energy: 27, fixed first mass 200 m/z). The isolation scheme consisted in 26 variable windows covering the 350–1650 m/z range with an overlap of 1 m/z^22^ (Supplementary Table 2).

#### DIA-MS data processing with Spectronaut

All DIA data was processed in Spectronaut version 14.10.201222.47784 (Biognosys) using direct DIA. Data were searched against the *Mus musculus* proteome with 58,430 protein entries (UniProtKB-TrEMBL), accessed on January 31, 2018. Trypsin/P was set as digestion enzyme and two missed cleavages were allowed. Cysteine carbamidomethylation was set as fixed modification, and methionine oxidation and protein N-terminus acetylation as variable modifications. Data extraction parameters were selected as dynamic, and nonlinear iRT calibration with precision iRT was selected. Identification was performed using a 1% precursor and protein q-value, and iRT profiling was selected. Quantification was based on the tandem MS peak area of the 3–6 best fragment ions per precursor ion, peptide abundances were obtained by summing precursor abundances and protein abundances by summing peptide abundances. Interference correction was selected, and local normalization was applied. Differential protein abundance analysis was performed using the paired *t* test, and *P* values were corrected for multiple testing, specifically applying groupwise testing corrections using the Storey^51^ method.^52^ For differential analysis, protein groups with at least 2 unique peptides and a q-value ≤ 0.05 were considered to be altered significantly (Supplementary Table 5).

### Bulk RNA Sequencing

Colon samples from three 10-month-old *Muc2*^*hom*^ and *Muc2*^*het*^ mice were used for bulk RNA sequencing. RNA extraction was performed following instructions from the Qiagen RNA extraction kit. Library preparation was performed by the genomics core at the Cancer Research UK Cambridge Institute using the Illumina Stranded messenger RNA Prep kit (20040532; Illumina) according to the manufacturer’s instructions. Samples were submitted for sequencing in the Illumina Novaseq platform with 50-bp paired-end reads. Differential expression analysis was performed using DESeq2^53^. An interaction model was used to identify differentially expressed genes in proximal or mid-*Muc2*^*hom*^ colon compared with location-matched *Muc2*^*het*^ samples.

### Bioinformatic Analysis

Plots were generated using either the ggplot2 package^54^ in R or GraphPad Prism.

#### Pearson correlation

The Pearson coefficients of correlation were determined between the different replicates using the stats package in R (version 4.0.2, version 1.3.1093; RStudio) and the abundances of all quantifiable protein groups as input.

#### Pathway analysis

An overrepresentation analysis was performed using ConsensusPathDB (release 35, 05.06.2021)^28^ to determine which Gene Ontology terms were enriched significantly. Gene Ontology terms identified from the overrepresentation or underrepresentation analysis were subjected to the following filters: q-value < 0.01, number of background genes ≥ 5, number of candidate genes ≥ 2, and log2 fold enrichment >1 or <-1.

#### Cell type deconvolution

Cell type signatures were created by combining single-cell RNA sequencing data sets provided in Kinchen et al,^40^ Smillie et al,^41^ and Mitsialis et al^42^ [DATASETS]. Two different data sets were created, with more- or less-specific cell types (Figures 7*F* and 9*B*). Significantly differentially expressed proteins (q-value < 0.05) or genes (from transcriptomic data, adjusted *P* value < 0.05) were mapped to cell type signatures and the spread of log2 fold changes for each cell type was used to extrapolate information on enrichment.

#### Protein interaction analysis

The ligand-receptor interaction set from the CellCellInteractions database (version 1.0, built April 25, 2017, containing iRefIndex version 14, Pathway Commons version 8, and BioGRID version 3.4.147)^35^ was used to find potential interactors to matrisomal proteins.

#### SLRP pathway enrichment

Pathway enrichment scores were calculated as follows: $\text{Pathway}\;\text{Enrichment}\;\text{Score}\;\left(\text{ES}\right)=\text{ES}\;\text{interactors}\times \left(\frac{N\;\text{differentially}\;\text{expressed}\;\text{interactors}}{N\;\text{interactors}}\right)$, where Enrichement Score^interactors^ is calculated as the average (log2 fold change) ∗ the average (q-value) for all interactors found in the pathway; N^diff^^erentially^ ^exp^^ressed^ ^interactors^ represents the number of interactors to SLRPs mapped to the pathway and expressed differentially in the relevant comparison; and N^interactors^ represents the total number of potential interactors to all SLRPs with interactors in the pathway.

#### Integrative network analysis

Protein parts of the colon matrisome^55^ and expressed differentially in the comparison of either proximal inflamed vs true normal or middle matched normal vs true normal were used for this analysis. Those data sets were processed to keep proteins annotated as ligands (Bader Laboratory data set of protein types). Bulk RNA sequencing performed on *Muc2*^*hom*^ vs *Muc2*^*het*^ colon samples was used to identify differentially expressed genes in the same comparisons. Those data sets were processed to keep genes annotated as receptors, and mapped to matrisomal ligands with which they have a described interaction.

#### Circos plots

The circlize package was used to produce circos plots,^56^ in which matrisomal proteins were aligned on the bottom and linked to potential interactors at the top, which were either mapped to pathways or cell types.
